# Robust p53 phenotypes and prospective downstream targets in telomerase-immortalized human cells

**DOI:** 10.18632/oncotarget.28690

**Published:** 2025-02-18

**Authors:** Jessica J. Miciak, Lucy Petrova, Rhythm Sajwan, Aditya Pandya, Mikayla Deckard, Andrew J. Munoz, Fred Bunz

**Affiliations:** ^1^Department of Radiation Oncology and Molecular Radiation Sciences, Sidney Kimmel Comprehensive Cancer Center, Baltimore, MD 21231, USA; ^2^Cellular and Molecular Medicine Graduate Program, The Johns Hopkins University School of Medicine, Baltimore, MD 21205, USA; ^*^These authors contributed equally to this work

**Keywords:** p53, ionizing radiation, immortalized cells, ALDH3A1, NECTIN4

## Abstract

Cancers that retain wild type *TP53* presumably harbor other clonal alterations that permitted their precursors to bypass p53-mediated growth suppression. Consequently, studies that employ *TP53*-wild type cancer cells and their isogenic derivatives may systematically fail to appreciate the full scope of p53 functionality. Several *TP53* phenotypes are known to be absent in the widely used isogenic HCT116 colorectal cancer (CRC) model, which originated from a tumor that had retained wild type *TP53*. In contrast, we show that restoration of p53 in the *TP53*-mutant CRC cell line DLD-1 impeded cell proliferation, increased levels of senescence and sensitized cells to ionizing radiation (IR). To study p53 in a non-cancer context, we disrupted *TP53* in hTERT-RPE1 cells. Derived from primary cells that were immortalized *in vitro*, hTERT-RPE1 expressed striking p53-dependent phenotypes and appeared to select for p53 loss during routine culture. hTERT-RPE1 expressed a p53-responsive transcriptome that was highly representative of diverse experimental systems. We discovered several novel downstream p53 targets of potential clinical relevance including *ALDH3A1*, which is involved in the detoxification of aldehydes and the metabolism of reactive oxygen species, and *nectin cell adhesion molecule 4* (*NECTIN4*) which encodes a secreted surface protein that is overexpressed in many tumors.

## INTRODUCTION

Genetic alterations that change the functions of p53 or other proteins in the p53 pathway are present in a majority of cancers. Despite the high frequency of *TP53* mutations, some tumor-derived cell lines retain wild type alleles and express wild type p53 protein. Such cell lines, paired with isogenic derivatives that are p53-deficient, have found wide use in basic and translational research. However, *TP53*-wild type cancer cells are not “normal”, nor can their p53 pathways be presumed to be entirely intact. On the contrary it is logical to assume that any expanding *TP53*-wild type cell population that enters the evolutionary bottleneck of tumorigenesis would require other compensatory clonal alterations to escape p53-mediated tumor-suppression.

Human somatic cells with targeted genetic modifications have contributed much to our understanding of human p53. The strengths and limitations of knockout cancer cell lines are exemplified by the first isogenic cell system for the study of human p53. Generated 25 years ago from the colorectal cancer cell line HCT116 [1] and widely distributed thereafter, the original human somatic cell p53-knockouts exhibit many cancer-relevant phenotypes, such as loss of cell cycle checkpoints and resistance to 5-fluorouracil (5-FU) [2], a first line therapeutic agent for CRC. This distinctive survival phenotype, uniquely elicited by 5-FU in p53-deficient HCT116 cells, established a plausible molecular mechanism for therapeutic resistance. However, HCT116 do not model the expected effects of p53 on growth in culture or on radioresistance [3]. Other basic processes such as p53 turnover, mediated by the feedback loop by which MDM2 controls p53 stability before and after DNA damage, are also defective in parental HCT116 [4].

In 2009, we employed targeted homologous recombination to derive additional p53-proficient and -deficient isogenic cell pairs from the CRC cell lines DLD-1, RKO, and SW48 [5]. This expanded cell panel from etiologically-related cancers was successfully used to identify new approaches to therapeutically exploit defects in p53. Like HCT116, each of these cell lines was derived from a mismatch repair-deficient cancer and therefore harbors many mutations [6]. Most of these mutations are undoubtably passengers of no functional consequence, but the full phenotypic impact of this high mutational burden remains unknown.

To study p53 in a human cell line with a low number of mutations and a defined basis for immortal growth, some investigators have turned to hTERT-RPE1. This cell line was derived from the primary retinal pigment epithelial cell line RPE-340 [7] Like other primary cells, RPE-340 undergo replicative senescence after 50–60 passages. This limit, first described by Hayflick, was successfully bypassed by the forced overexpression of the catalytic subunit of the enzyme telomerase, hTERT. Thus immortalized, hTERT-RPE1 cells have a stable diploid karyotype, are non-tumorigenic [8] and are widely used as a model of normal cellular function in studies of cell signaling and cell proliferation.

In most cell lineages, the bypass of replicative senescence requires the introduction of a cellular or viral oncogene, such as SV40 large T antigen, in addition to telomerase. Such oncogenes typically interact with p53 and suppress its function. hTERT-RPE1 cells, immortalized by telomerase expression alone, are therefore uniquely suited for the study of p53 phenotypes.

Studies employing hTERT-RPE1 cells and CRISPR-based gene editing techniques have recently provided new insight into some of functions of p53 that are important during unperturbed growth, such as the arrest of cell growth in response to mitotic dysfunction [9] and the suppression of polyploidization [10]. Some relatively subtle p53 phenotypes observed in hTERT-RPE1 are notably lacking in p53-proficient cancer cells. For example, Solokova et al. [11] observed that slowing the rate of DNA replication by histone depletion caused a p53-dependent cell cycle arrest in hTERT-RPE1 cells. This response was reportedly absent in cancer cell lines such as HCT116, which were found to more closely resemble p53-deficient hTERT-RPE1 in this regard.

Indirect evidence also points to a particularly robust p53 pathway in non-cancer cell types. Several groups have reported that the small numbers of targeted double strand DNA breaks created by CRISPR-Cas9 complexes are sufficient to activate p53 in human stem cells and hTERT-RPE1 [12, 13]. In the context of a CRISPR library screen, the activation of p53 can markedly reduce the yield, as many clones are stochastically eliminated. This technical issue, particularly problematic in non-cancer models, can reportedly be overcome by modified screening protocols [14] but nonetheless indicates a sensitivity to p53 activation that is apparently lacking in many cell types.

Senescence is an important barrier to neoplastic cell growth and an antiproliferative response to p53 activation. We previously observed that senescence could not be induced in HCT116 cells by DNA damage unless they were haploinsufficient for *hTERT* [3], which encodes the catalytic subunit of telomerase. One interpretation of this finding is that the pathways to senescence remain partially intact in HCT116, but were somehow downregulated during tumorigenesis. Here we report that restoration of wild type p53 in DLD-1, a p53-deficient CRC cell line, was sufficient to cause elevated levels of senescence and sensitivity to IR. In addition, our comprehensive analysis of non-senescing hTERT-RPE1 cells revealed prominent p53-dependent phenotypes, and novel downstream targets and pathways to be further explored.

## RESULTS

### Restoration of p53 in DLD-1 cells reduces proliferation and increases senescence

**Figure 1:**
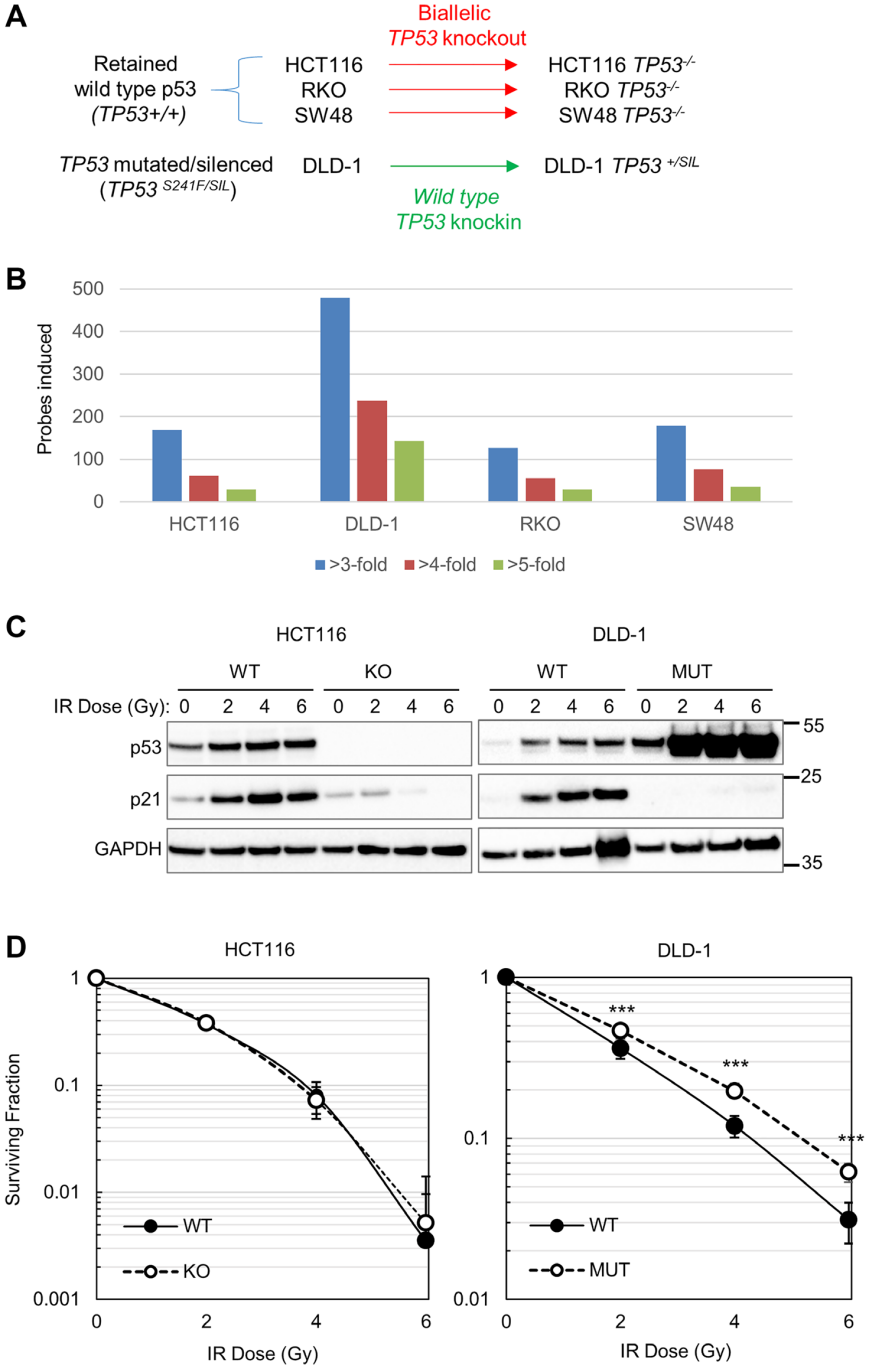
p53-dependent responses to ionizing radiation in CRC cells. (**A**) The schema for derivation of isogenic CRC cell pairs by *TP53* knockout or knockin, as reported in (5). (**B**) Gene expression data from unperturbed and irradiated CRC cells were downloaded from the Gene Expression Omnibus (GEO) database (GSE13886). The data were first filtered to include only probe signals that were induced >2-fold by IR. Values shown are the respective numbers of unique array probes that were upregulated in *TP53*-wild type cells compared with isogenic *TP53*-knockout or mutant cells at the indicated cutoffs. (**C**) HCT116 and DLD-1 cells and their respective isogenic derivatives were treated with the indicated doses of X-rays and harvested after 24 h. The indicated proteins in the resulting cell lysates were quantified by western blot. The migration of relevant molecular weight markers, in kDa, are shown to the right of each blot. (**D**) Clonogenic survival of HCT116 and DLD-1 cell and their isogenic derivatives following treatment with X-rays at the indicated doses. Each data point is the average fractional survival from three plates; error bars represent the standard deviation. Three asterisks (^***^) indicates *p* < 0.0001, as determined by a two-sample *t*-test. No asterisks, *p* < 0.1, deemed not significant.

In response to DNA damage, p53 directly stimulates the transcription of numerous downstream target genes [15, 16]. To comparatively evaluate p53-dependent responses to IR in cell lines originating from the same tissue, we examined four isogenic CRC cell pairs previously generated by homologous recombination [5]. Three of the parental cell lines, HCT116, RKO and SW48, harbor wild type *TP53*; these wild type alleles were disrupted to create respective p53-deficient knockouts in each line (Figure 1A). A fourth cell line, DLD-1, exclusively expresses a mutant form of p53 in which the serine residue at position 241 is replaced by phenylalanine (S241F). Wild type p53 function was restored in these cells by knocking in wild type coding sequences into the endogenous locus [5].

We reevaluated DNA damage- and p53-dependent gene expression in this CRC cell panel. To quantify the number of transcripts that were induced by p53 in response to IR, we first identified microarray probe signals that were induced more than 2-fold by IR in each p53-proficient cell line, as described [5]. Among each IR-responsive probe set, we next determined how many were induced at a higher level in p53-proficient versus p53-deficient cells of the same type. The DLD-1 isogenic cell pair, created by p53 restoration, produced the largest number of IR-induced, p53-dependent probe signals (Figure 1B); each of the cell lines that naturally retained wild type *TP53* induced a lower number of probes at each cutoff.

p53 and its canonical downstream target p21 were induced by IR in HCT116, the most widely disseminated among the cells in this CRC panel, and in DLD-1 with restored p53 function (DLD-1 p53^WT^) (Figure 1C). Both p53 and p21 appeared to be induced to a greater extent in DLD-1 p53^WT^ than in HCT116 p53^WT^, which expressed detectable levels of both proteins in the absence of IR treatment. Consistent with previously published results from our laboratory, there was no significant difference in clonogenic survival after IR treatment between p53-proficient and -deficient HCT116 cells (Figure 1D). In contrast, DLD-1 p53^WT^ cells were demonstrably radiosensitive when compared with isogenic DLD-1 p53^MUT^ cells.

**Figure 2:**
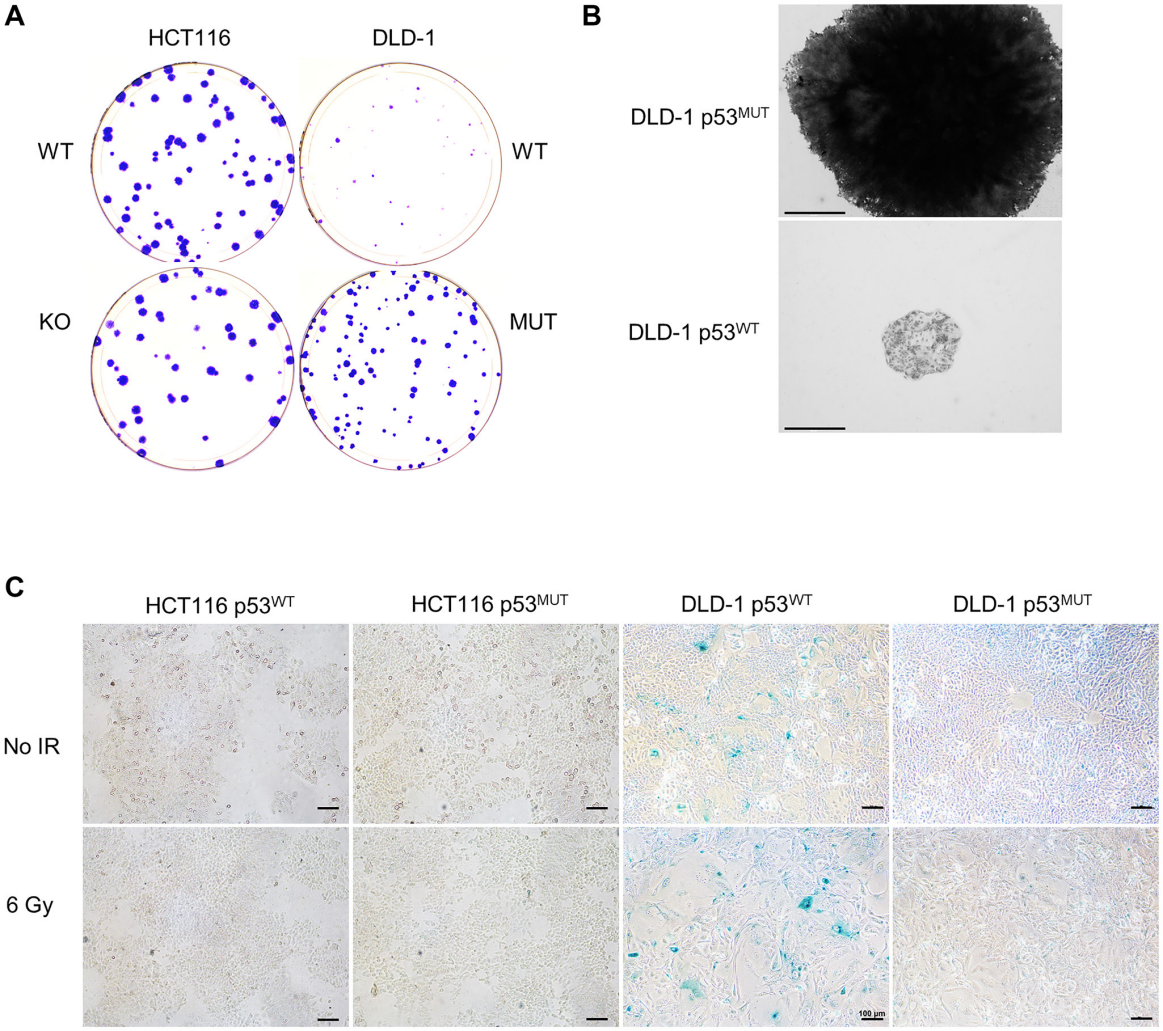
Growth of DLD-1 cells with restored p53 function. (**A**) Representative control plates (no IR treatment) from the clonogenic survival assays show in Figure 1D, stained with crystal violet. (**B**) Representative colonies imaged at 4X magnification. Scale bars = 650 µm. (**C**) Monolayer cultures treated with a single 6 Gy dose of X-rays, and untreated cultures, were fixed with glutaraldehyde 24 h later and stained for senescence-associated β-galactosidase under 10X magnification. Scale bars = 100 µm.

The colonies formed by DLD-1 p53^WT^ on all plates, irrespective of their treatment, were smaller than those formed by isogenic cells that expressed mutant p53 (Figure 2A, 2B), suggesting that p53 was an impediment to cell proliferation. Monolayer cultures were stained for senescence-associated β-galactosidase (Figure 2C). Despite the low level of p53 protein present in non-irradiated cells (Figure 1C), DLD-1 p53^WT^ cells stained positive for this marker of senescence; staining appeared more pronounced following IR treatment. In contrast, few stained cells were observed in the DLD-1 p53^MUT^ cultures or in HCT116 of either genotype.

### Increased cell proliferation following disruption of TP53 in hTERT-immortalized cells

We next sought to investigate the effects of p53 in human cells that did not originate in a tumor. Our rationale was that in the absence of *in vivo* selection for loss of p53 function, immortalized cells might retain more subtle but quantifiable p53 phenotypes that are commonly lost in evolving cancers.

**Figure 3:**
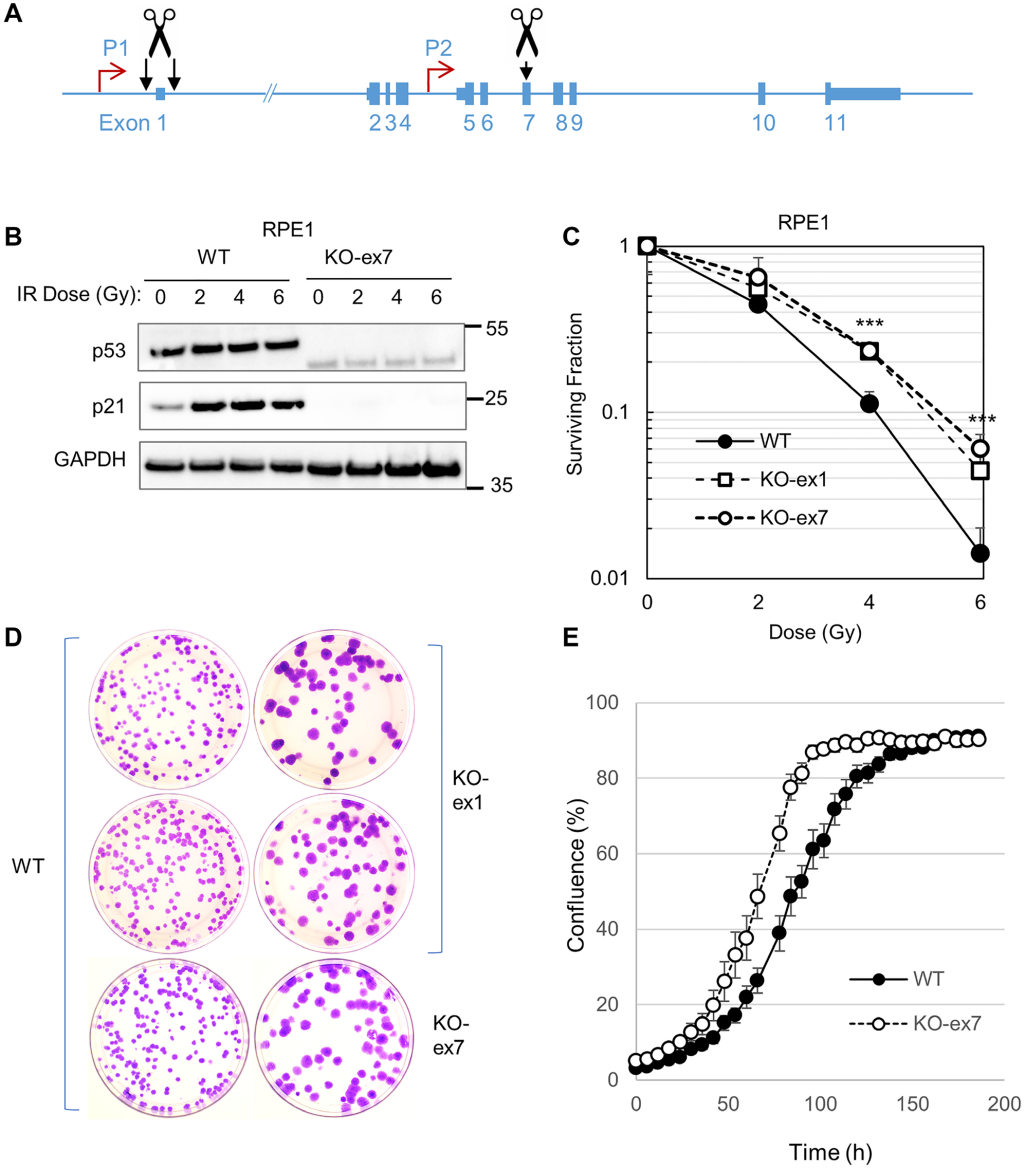
Disruption of the *TP53* locus in hTERT-RPE1 cells. (**A**) SpCas9 guide RNAs were designed to target protospacer sequences in the *TP53* gene (shown in blue). A single site was targeted in exon 7; a dual guide approach was used to delete *TP53* exon 1. (**B**) hTERT-RPE1 cells and the two isogenic knockouts in exon 1 (KO-ex1) or exon 7 (KO-ex7) were treated with the indicated doses of X-rays and harvested 24 h later. The indicated proteins were quantified by western blot. The migration of relevant molecular weight markers, in kDa, are shown to the right of each blot. (**C**) Clonogenic survival of hTERT-RPE1 and p53-knockout derivatives following treatment with X-rays at the indicated doses. Each data point is the average fractional survival on three plates; error bars represent the standard deviation. Three asterisks (^***^) indicates a value of *p* < 0.0001 between the wild type cells and each of the p53-knockouts, as determined by two-sample *t*-tests. No asterisks, *p* < 0.1, deemed not significant. The differences between the two independent p53-knockout clones were not significant (*p* > 0.1). (**D**) Approximately 200 cells of the indicated genotypes were plated to 10 cm plates, which were then incubated for 14 d and stained with crystal violet. (**E**) To measure cell growth, 1000 cells were plated in a 96-well plate in triplicate. Cell density was recorded every 6 h by an Incucyte imaging system.

The *TP53* locus expresses several related proteins from two endogenous promoters (Figure 3A). To eliminate the possible confounding effects of off-target editing, we eliminated p53 expression by two editing approaches. *TP53* exon 7, which is common to all known isoforms, was disrupted at a single site. Alternatively, we simultaneously targeted two sites that flank exon 1, thereby causing a deletion. Multiple knockout clones were identified by western blot and confirmed by Sanger sequencing.

As expected, p53 and p21 were induced in hTERT-RPE1 cells by IR (Figure 3B). Both of the p53 knockout cell lines, KO-ex7 and KO-ex1, were radioresistant compared with p53-proficient parental cells (Figure 3C). As in the DLD-1 isogenic system (Figure 2A), colonies that expressed wild type p53 were noticeably smaller than those formed by p53-knockout cells (Figure 3D). The size of the colonies was fairly uniform within each population, suggesting that there was limited variation between subclones of the same genotype. An increased rate of growth in p53 knockout cells was additionally quantified by time lapse microscopy (Figure 3E).

### Expansion of an hTERT-RPE1 subclone that harbors a cancer-associated p53 mutation

**Figure 4:**
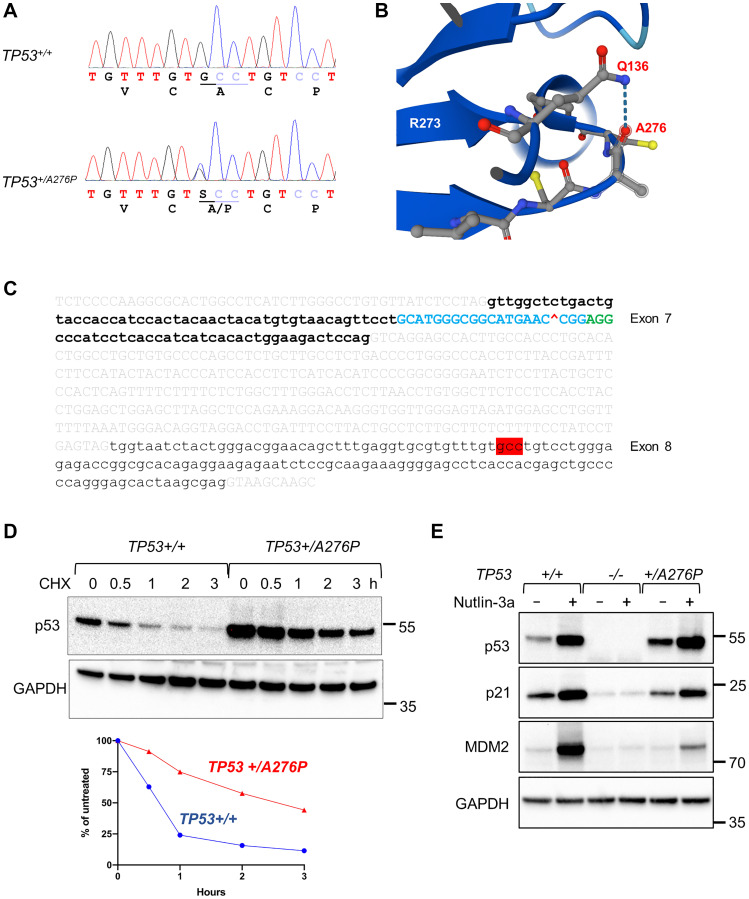
Identification of a *TP53^+/A276P^* subclone. (**A**) Sanger sequence traces of wild type *TP53* in the bulk cell population and a heterozygous C→T mutation, identified in a single clone. (**B**) A structural analysis predicts a stable hydrogen bond between A276 and Q136. The rendering generated by AlphaFold was based on 245 structures available in UniProt P04637. (**C**) The genomic sequence containing *TP53* exons 7 and 8 is shown. Intron sequences are indicated by light gray text. The CRISPR/Cas guide sequence template in exon 7 is shown in blue text, the predicted cut site is indicated with a red “^” symbol, and the protospacer adjacent motif is shown in green. Codon 276, located in exon 8, is highlighted in red. (**D**) Cells were continuously treated with 100 µg/ml cycloheximide and harvested at the indicated time points. Protein extracts were probed for p53 and GAPDH (upper panel). Levels of protein were quantified by densitometry (lower panel). (**E**) Wild type hTERT-RPE1 cells, the *TP53* exon 7 knockout, and the TP53^+/A276P^ mutant clone were untreated or treated with 10 µM nutlin-3a for 8 h. Protein extracts were probed for the indicated proteins. The migration of relevant molecular weight markers, in kDa, are shown to the right of each blot.

We inadvertently isolated a single clone that retained wild type exon 7 sequences and expressed elevated levels of p53 protein. We sequenced the remaining exons in this clone and identified a single nucleotide substitution in exon 8 (Figure 4A). A heterozygous C-to-G transversion changed the encoded amino acid from an alanine residue at position 276 to proline (A276P). This alanine residue is predicted to form a hydrogen bond with Q136 (Figure 4B), and thus may contribute to structural stabilization. Codon 276 is located 427 bp from the predicted CRISPR cut site in exon 7 (Figure 4C). No other base alterations were noted.

A276P mutations were found in 19 tumors profiled in the TCGA database (Table 1). Overall, A276P/D/G mutations were reported in a relatively small number of tumors from diverse tissues [17, 18]. We expanded the heterozygous *TP53^+/A276P^* clone so that we could evaluate the phenotypic impact of this cancer-associated mutation.

**Table 1:** Somatic *TP53* c.826G>C (A276P) mutations

Sample_Name	Sample_ID	Morphology	Sex/age	Study PMID
NY98-JP6	8713	Gallbladder adenocarcinoma	F/70	9568784
CM96-LA17	7277	Osteosarcoma	NA	8781571
MOU96-333-1	5367	Liposarcoma	NA	8821948
KAN96-7	5138	Burkitt lymphoma	M/12	9172810
TAT95-1	4781	Thymoma, NOS	M/48	7572785
TAT95-10	4782	Thymoma, NOS	F/44	7572785
TAT95-2	4785	Thymoma, NOS	F/51	7572785
HCH94-35	2889	Hepatocellular carcinoma	NA	21567059
HV11	2265	Rectal adenocarcinoma	M/46	8317886
Su1	764	Precursor B-cell lymphoblastic leukemia	NA	1705829
Co-12	1121	Breast cancer, NOS	F	1394133
Su-1	902	Renal cell carcinoma, NOS	NA	1582882
ICH00-GB28	12438	Glioblastoma multiforme	NA	10667596
LUK00-C425	13747	Papillary carcinoma in situ	F	10623666
RMT98-1	9748	Chondrosarcoma	NA	9646035
DOL03-51	18386	Barrett’s esophagus	M	12823203
TCGA-13-0807	27778	Ovarian, serous cystadenocarcinoma	F	21720365
PAC07-4	26430	Basal cell carcinoma	NA	18070208
PIJ06-R25	22739	Endometrioid adenocarcinoma	F	16271749

A cycloheximide chase experiment demonstrated that p53 in the hTERT-RPE1 *TP53^+/A276P^* cells was abnormally stable (Figure 4D), a cardinal feature of many tumor-associated p53 mutant proteins. The abundance of p53 is controlled by a feedback loop involving the E3 ubiquitin ligase MDM2, which is induced by p53 and targets p53 for degradation by the proteosome. As expected, treatment of parental hTERT-RPE1 cells with the MDM2 inhibitor nutlin-3a caused p53 stabilization and robust induction of p21^WAF1/CIP1^ and MDM2 (Figure 4E) [16, 19]; these downstream effectors were not induced in the p53-knockouts. p21^WAF1/CIP1^ and MDM2 were induced to a lesser extent in *TP53^+/A276P^* cells compared with wild type cells. The simplest interpretation of this observation was that the p53^A276P^ mutant protein was exerting a dominant negative effect on the co-expressed p53-wild type protein.

The spontaneous expansion of a cancer-associated p53 mutation in a cultured cell population is an extremely rare event. In more than 25 years of study, we had never previously detected a heterozygous *TP53*-mutant subclone. As there are few heterozygous cellular models that co-express wild type p53 and a cancer-associated mutant, we decided to use this unique model to characterize a wide range of p53-dependent phenotypes. Our goals were (1) to establish which of the many reported p53-dependent phenotypes could be elicited in the hTERT-RPE1 cell line, and (2) to explore which of these phenotypes was subject to dominant-negative inhibition by the p53^A276P^ mutant protein.

### The dominant-negative effect of p53^A276P^ appears to be limited to transcriptional transactivation

While activated p53 upregulates the activity of its target genes after DNA damage or MDM2 inhibition, p53 suppresses transcription in the absence of upstream signals [20, 21] . The epigenetic repression of transcription by p53 is mediated by the methylation of histone H3K9 [22, 23]. A repressive mark, H3K9 trimethylation is continuously maintained in the absence of DNA damage by a chromatin-bound complex containing p53, USP7 and MDM2, which cooperatively recruits the histone methylase SUV39H1 [24]. This complex is rapidly disassembled following DNA damage and the resulting stabilization of p53. With local chromatin in an active euchromatic state, p53 forms DNA-bound tetramers that are required for target gene induction. Conversely, the H3K9me3 mark is elevated in heterochromatin, and maintained in this transcriptionally inactive state by monomeric and dimeric p53. By exerting dynamic control over select genes before and after DNA damage, p53 mediates a bistable transcriptional switch.

**Figure 5:**
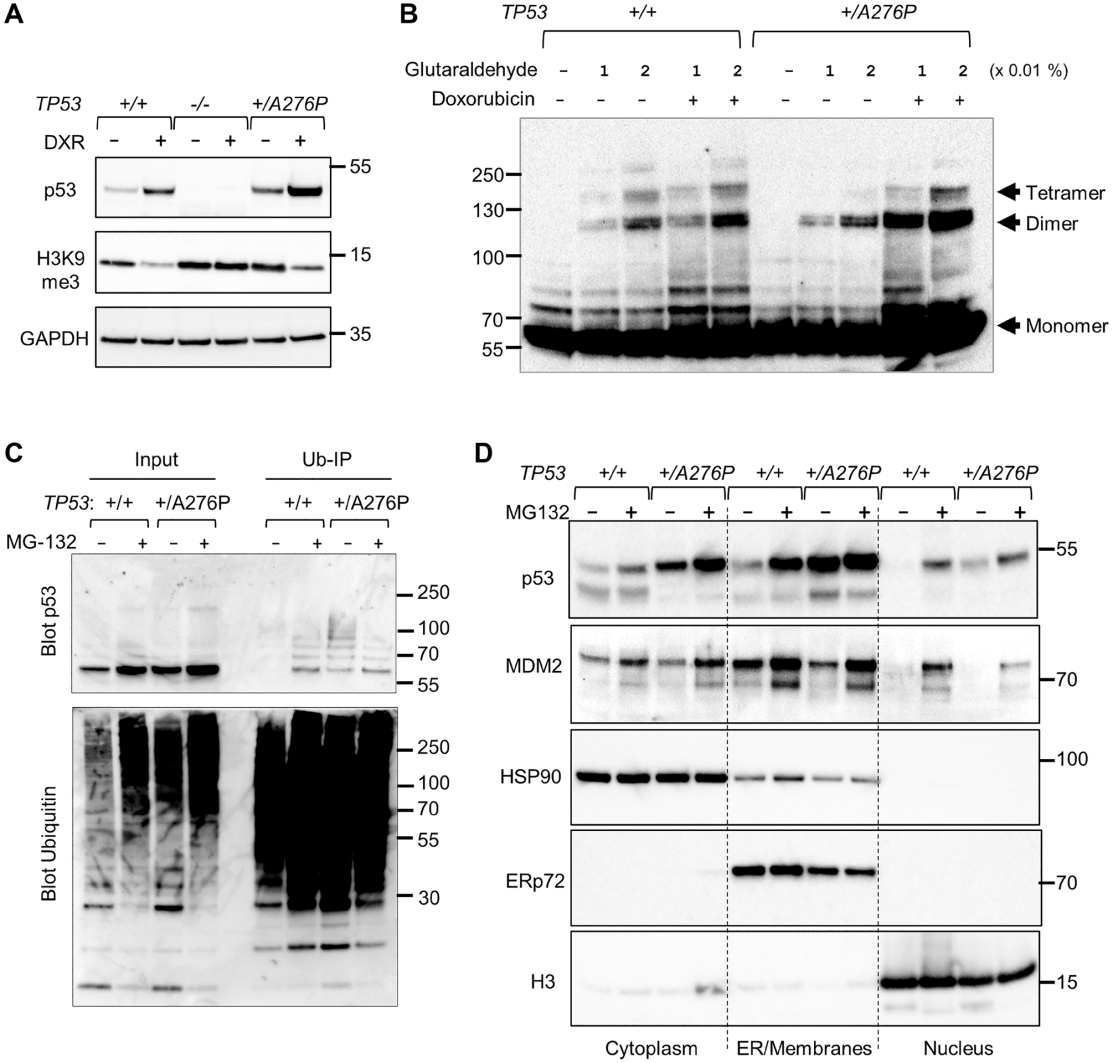
Impact of p53 A276P mutant on oligomerization and subcellular localization. (**A**) Unmodified hTERT-RPE1 cells, a *TP53* exon 7 knockout clone and the *TP53^+/A276^* clone were treated with 0.2 µg/ml doxorubicin for 48 h. Extracts were probed for the indicated proteins. (**B**) Untreated cells and cells treated for 24 h with 0.2 µg/ml doxorubicin were lysed and treated with either 0.01% or 0.02% glutaraldehyde, as indicated. Crosslinked oligomers were detected on a western blot probed with a monoclonal antibody against p53. (**C**) Untreated cells and cells treated with the proteasome inhibitor MG-132 (10 µM) for 4 h were lysed. Ubiquitinated proteins were pulled down as described in Experimental procedures. Equal amounts of lysate (input) and bead eluate were fractionated and probed for p53 or ubiquitin, as indicated. (**D**) Cells treated as in (A) were fractionated into cytoplasmic, intracellular membrane, and nuclear components. Fraction-specific proteins were probed with antibodies against MDM2 and p53. HSP90, ERp72 and histone H3 were detected on a separate blot run in parallel to assess protein recovery. The migration of relevant molecular weight markers, in kDa, are shown to the right of each blot.

The chemotherapeutic agent doxorubicin stimulates p53 tetramerization and thereby disrupts the complex required for the retention of SUV39H2 at p53 responsive promoters [23]. A global reduction in H3K9me3 protein following doxorubicin treatment, previously characterized in the colorectal cancer cell line HCT116 [23], was clearly observed in unmodified hTERT-RPE1, retained in the *TP53^+/A276P^* line but completely absent in p53-deficient hTERT-RPE1 cells (Figure 5A).

We further examined the effects of the p53^A276P^ mutant on p53-dependent gene regulation by assessing oligomeric, chromatin-associated complexes of endogenous p53 proteins. By crosslinking these protein complexes with glutaraldehyde, we were able to resolve endogenous p53 dimers, tetramers and higher order oligomers (Figure 5B). In the absence of DNA damage, most of the p53 complexes in *TP53*^+*/A276P*^ cells were dimers, which are generally transcriptionally inactive. Cells that expressed only wild type p53, in contrast, exhibited a range of p53 complexes, including transcriptionally active tetramers. Following DNA damage, p53 in wild type cells formed both dimers and tetramers. *TP53^+/A276P^* cells expressed a proportionally lower amount of tetrameric p53, consistent with the observed reduction in p21 and MDM2 induction in this line (Figure 4E). These patterns suggest that the stable p53^A276P^ mutant protein primarily formed repressive homo- and heterodimers, consistent with a dominant-negative effect on transcription.

The localization of p53 is tightly controlled by ubiquitination. In the absence of DNA damage, p53 largely resides outside the nucleus. In unstimulated cells with low p53 and low MDM2, the nuclear export of p53 is mediated by MDM2-mediated mono-ubiquitination [25]. When MDM2 levels are high, as is the case when cells are recovering from DNA damage, p53 is polyubiquitinated and thus rapidly targeted for degradation by the proteasome. Pulldowns of ubiquitin revealed increased levels of p53 mono- and poly-ubiquitination in the unperturbed *TP53^+/A276P^* cells (Figure 5C). Accordingly, p53 was found disproportionately in the membrane-associated fraction in *TP53^+/A276P^* (Figure 5D). The basal levels of nuclear p53 were more modestly increased in these cells compared with parental hTERT-RPE1.

**Figure 6:**
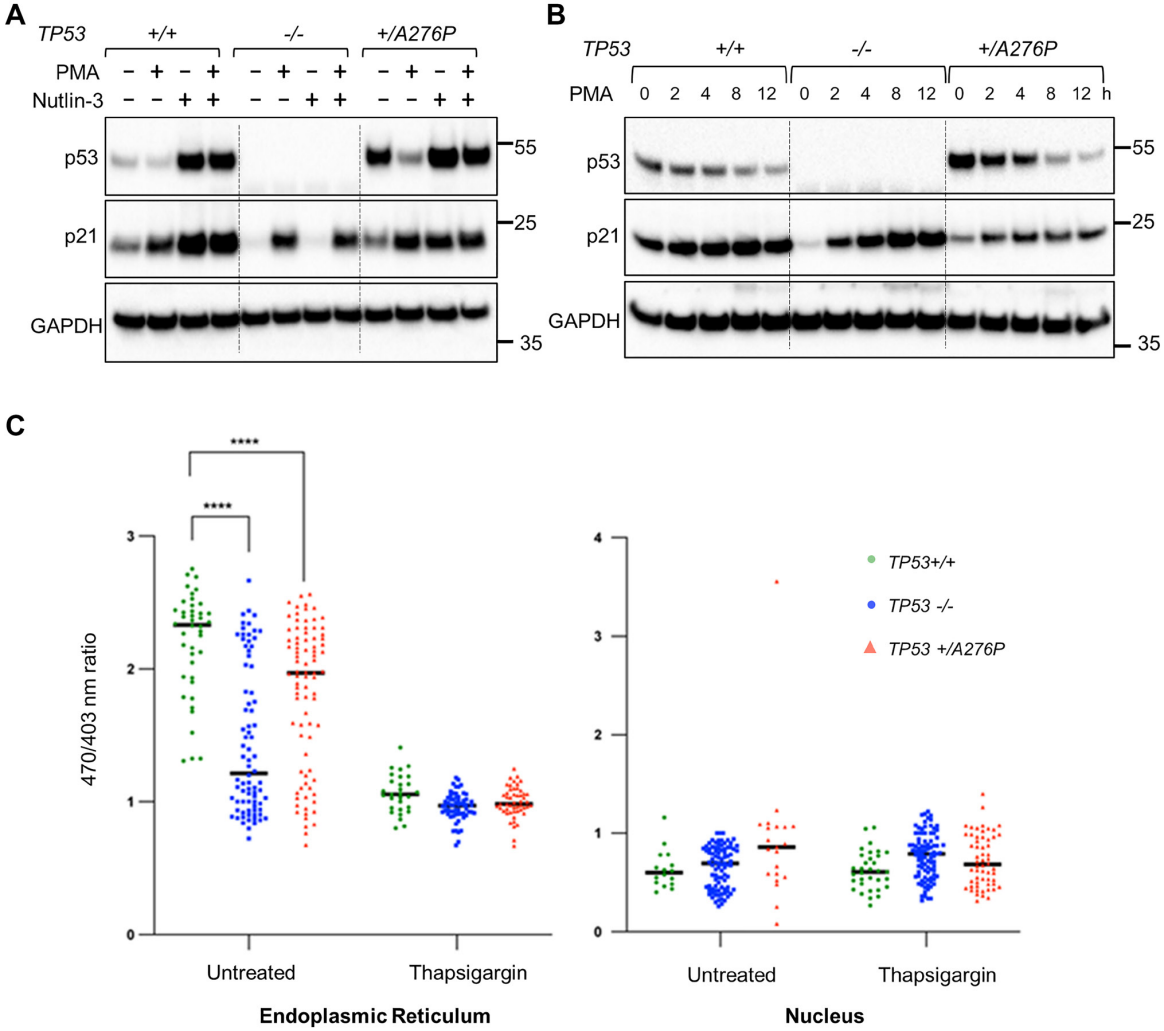
Upstream and downstream effects of p53 on intracellular Ca^2+^ signaling. (**A**) Monolayer cell cultures were treated with nutlin-3a (10 µM) and/or PMA (100 ng/ml) for 8 h. (**B**) Cells were treated with 100 ng/ml PMA and harvested for protein analysis at the indicated time points. The indicated proteins were assayed by western blot. The migration of relevant molecular weight markers, in kDa, are shown to the right of each blot. (**C**) GAP biosensors were used to assess relative Ca^2+^ levels in the ER and the nucleus. Where indicated, ER Ca^2+^ was selectively depleted by treating cells with the SERCA inhibitor thapsigargin for 24 h. The *TP53*^−/−^ cells used in these experiments contain the exon 7 knockout.

Phosphorylation by calcium-dependent protein kinase C (PKC) has also been identified as an important requirement for normal p53 turnover in unstressed cells [26, 27]. To stimulate p53 turnover via PKC, we treated hTERT-RPE1 cells with phorbol 12-myristate 13-acetate (PMA). This phorbol ester is a synthetic analog of diacyl glycerol, the endogenous activator of PKC-mediated signal transduction. *TP53*^+/+^, *TP53*^−/−^ and *TP53^+/A276P^* cells were treated with PMA alone, or with PMA in combination with nutlin-3a (Figure 6A). PMA did not affect the stabilization of p53 after MDM2 inhibition, supporting the current model in which MDM2 and PKC work in concert to control p53 turnover in unstressed cells. However, PMA administered without nutlin-3a selectively and dramatically decreased the level of p53 in the *TP53^+/A276P^* cell line. Over a 12 h time course, PMA reduced p53 to similar levels in *TP53*^+/+^ and *TP53^+/A276P^* cells (Figure 6B).

PMA has antiproliferative effects on some cell types, which are mainly mediated by the p53-independent induction of p21^WAF1/CIP1^ [28–30]. This Ca^2+^ dependent pathway for cell cycle regulation was clearly intact in the hTERT-RPE1 cell panel (Figure 6A, 6B). Interestingly, the p53-independent upregulation of p21^WAF1/CIP1^ by PMA was attenuated in *TP53^+/A276P^* cells (Figure 6B). It is possible that p53 dimers containing mutant protein (Figure 5B) enhanced epigenetic silencing at the *CDKN1A* promoter in the absence of DNA damage. These experiments illustrate how the turnover of wild type p53 and the p53^A276P^ mutant are similarly controlled by Ca^2+^-dependent signaling, suggesting a possible therapeutic approach to suppressing the gains-of-function caused by this mutation.

As an important mediator of apoptosis, p53 also plays a cytoplasmic role in Ca^2+^ signaling. p53 directly binds to the Sarcoendoplasmic Ca^2+^-ATPase (SERCA) pump at the endoplasmic reticulum (ER) and mitochondria-associated membranes and stimulates the enhanced transfer of Ca^2+^ to the mitochondria [31]. In this transcription-independent manner, wild type p53 increases mitochondrial outer membrane permeability and lowers the threshold for apoptosis.

The abundance of wild type and mutant p53 at the ER-associated membranes of hTERT-RPE1 cells (Figure 5D) prompted us to investigate the impact of these proteins on SERCA activity. We used a genetic biosensor to measure the relative levels of Ca^2+^ in the ER lumen, the main repository for intracellular calcium, and in the nucleus. An aequorin-based Ca^2+^ reporter system, insensitive to local pH and Mg^2+^, can be specifically targeted to several organelles via fusion with signaling peptides [32]. Parental hTERT-RPE1, *TP53*^−/−^ and the *TP53^+/A276P^* mutant cell line were each stably transfected with reporter constructs encoding a green fluorescent protein (GFP)-Aequorin fusion Protein (GAP) targeted to the ER or to the nucleus. Organelle-specific Ca^2+^ levels were then determined in individual cells by dual-excitation ratiometric imaging, as described [32].

The levels of Ca^2+^ in the ER were significantly reduced in *TP53*^−/−^ compared with wild type cells; Ca^2+^ levels were decreased to an intermediate level in *TP53^+/A276P^* cells (Figure 6C). This finding is consistent with the established role of cytoplasmic p53 as a direct SERCA activator [31]. As the levels of ER-localized p53 were significantly elevated in the *TP53^+/A276P^* cell line (Figure 5D), we infer that the mutant p53^A276P^ protein was non-functional with respect to SERCA activation, but did not exert a dominant negative effect. As expected, the store of Ca^2+^ in the ER was depleted in response to SERCA inhibition by thapsigargin in all cells irrespective of genotype. The levels of Ca^2+^ in the nucleus were predictably low and did not differ substantially between the three isogenic cell lines (Figure 6C).

In summary, the dominant-negative effects of p53^A276P^ are most clearly manifest by the inhibition of transcriptionally active p53 complexes. Interestingly, this inhibitory effect was also apparent at the p21 promoter when it was activated by a p53-independent mechanism (Figure 6A, 6B). In contrast, the p53^A276P^ protein did not appear to inhibit the cytoplasmic function of wild type p53 at SERCA (Figure 6C). Luminal Ca^2+^ was somewhat decreased in the *TP53^+/A276P^* cells, but we believe this observation can be most likely attributed to reduced *TP53* gene dosage rather than a dominant-negative effect.

### Induction of p53 target genes is variably inhibited by p53^A276P^

Cancer cells that harbor inactivating mutations in *TP53* express only the corresponding p53 mutant protein; wild type protein expression is invariably lost via loss-of-heterozygosity (LOH) or gene silencing, as in DLD-1. The heterozygous hTERT-RPE1 cell clone therefore provided a unique opportunity to evaluate the functional impact of p53^A276P^ when co-expressed with wild type protein. To quantify such effects, we evaluated gene expression.

**Figure 7:**
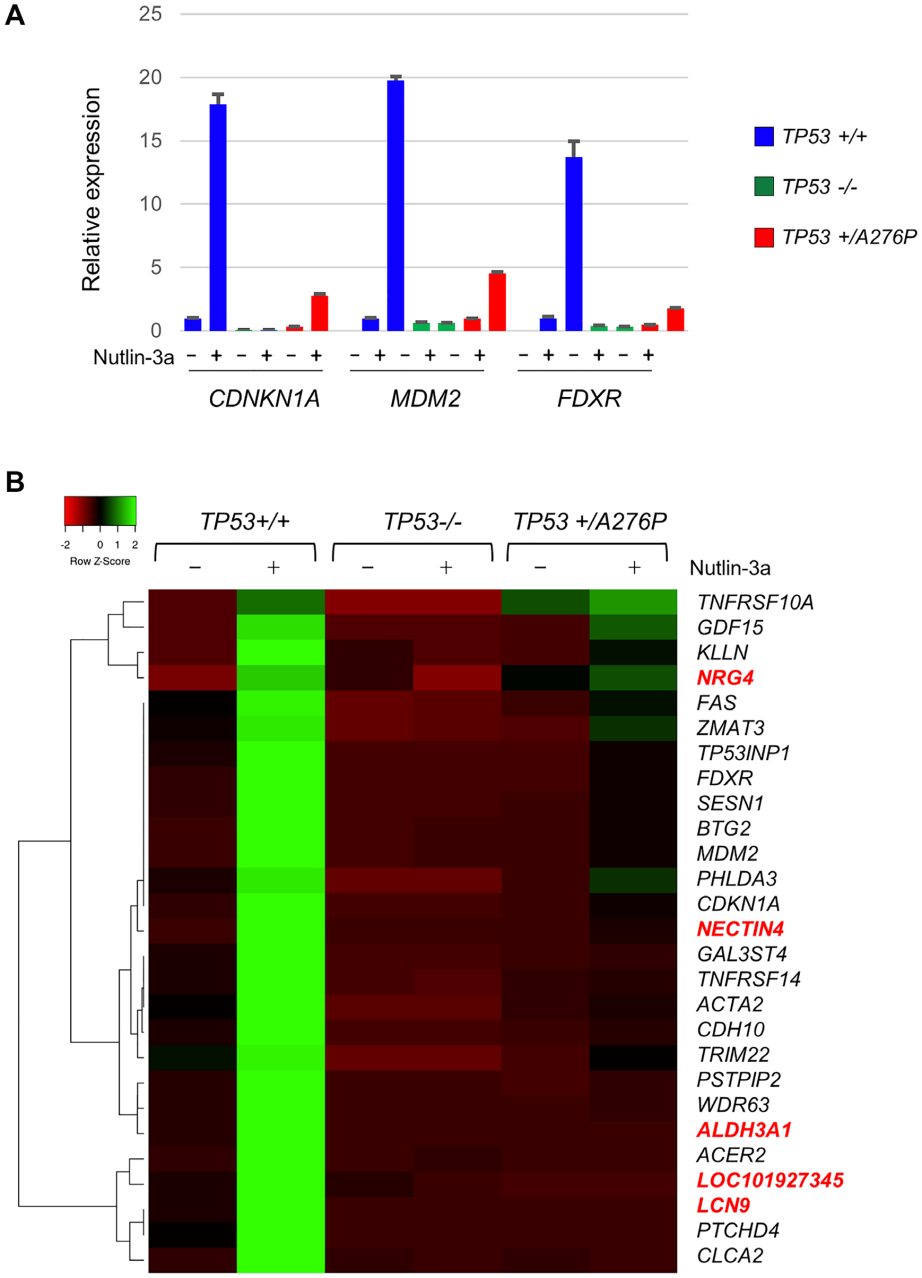
Dominant attenuation of p53-dependent gene expression by p53 A276P. (**A**) The induction of the known p53 target genes *CDKN1A*, *MDM2* and *FDXR* following treatment with 10 µM nutlin-3a for 8 h was assessed by RT-qPCR. For each sample, target gene expression was normalized to a *GAPDH* control and the relative expression was calculated in comparison with untreated parental hTERT-RPE1. (**B**) RNA-seq analysis of the p53-dependent transcriptome in hTERT-RPE1 and indicated derivatives. A heatmap illustrates the clustered relationships between genes that were induced at least 3-fold by nutlin-3a in the *TP53*^+/+^ hTERT-RPE1 cells, and that were also upregulated by at least 10-fold in the nutlin-3a-treated wild type cells over the identically-treated *TP53*^−/−^ cells (exon 7 knockout). Genes highlighted in red were not previously identified as p53 transcriptional targets. The RT-qPCR results shown are representative of three independent experiments.

The expression of several established p53 targets was first confirmed by RT-qPCR. As expected, *CDKN1A* (which encodes p21), *MDM2* and *FDXR* were each robustly induced in unmodified hTERT-RPE1 following stimulation with nutlin-3a (Figure 7A). Induction of these genes was lost in *TP53*^−/−^ cells and notably reduced in the *TP53^+/A276P^* cells, demonstrating a dominant negative effect of the p53^A276P^ mutant protein with respect to transcriptional transactivation.

We next characterized the p53-dependent transcriptome expressed in hTERT-RPE1 by bulk RNA-seq (Figure 7B). A set of genes that were tightly controlled by p53 in these cells was defined by first identifying those that were induced at least 3-fold in wild type hTERT-RPE1 after treatment with nutlin-3a for 8 h. This early time point was chosen to minimize indirect effects caused by upregulation by p53 of other transcription factors. Among these nutlin-3a responsive genes, 27 were induced at least 10-fold higher in wild type RPE1 cells compared with isogenic *TP53*^−/−^ cells that were treated with nutlin-3a in parallel. The induction of this defined transcriptome by nutlin-3a was broadly attenuated in the heterozygous *TP53^+/A276P^* cells (Figure 5B).

### Identification and validation of novel p53 target genes

Of the 27 genes most robustly upregulated in a p53-dependent manner, 22 had previously been linked to p53 with varying levels of confidence in disparate experimental systems [33–58]. The remaining five genes had not previously been identified as transcriptional targets of p53. To investigate whether their regulation might involve direct transactivation by p53, we examined publicly available chromatin immunoprecipitation (ChIP)-seq data in the ReMap2022 database [59]. Among these five prospective p53-regulated genes identified in our analysis, two harbored p53 binding sites that were experimentally identified.

**Figure 8:**
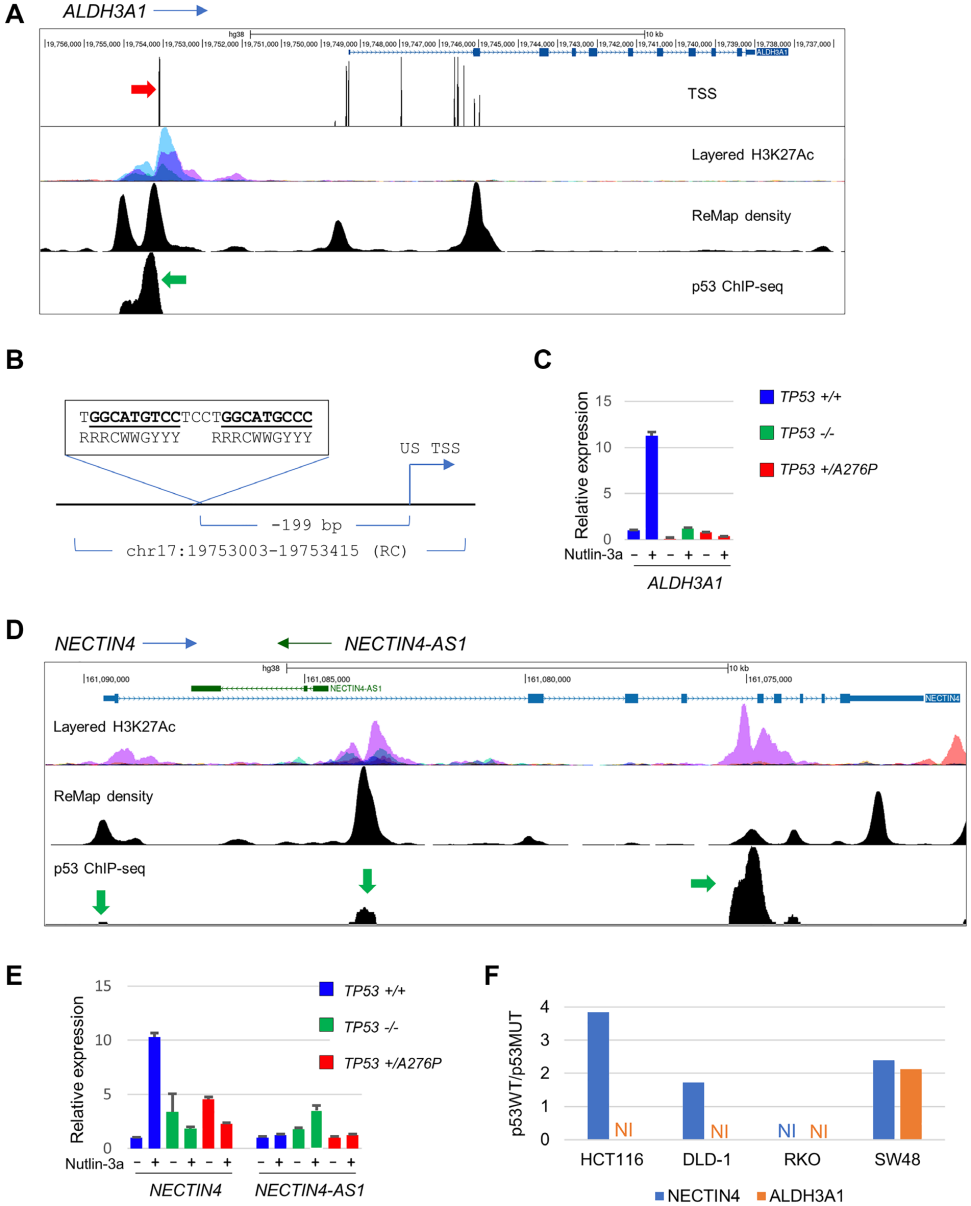
Regulation of ALDH3A1 and NECTIN4 by p53. (**A**) The indicated tracks showing H3K27Ac density data on 7 cells lines from ENCODE, ChIP-seq density and p53-specific ChIP-seq data from ReMap and putative transcription start sites identified via CAGE by the FANTOM5 consortium are shown below a schematic of the ALDH3A1 locus. An upstream p53 ChIP-seq peak is indicated by the green arrow; the associated TSS is indicated by a red arrow. (**B**) A putative p53 response element was found -199 bp upstream of the TSS. (**C**) RT-qPCR analysis of relative ALDH3A1 expression across the hTERT-RPE1 cell panel. (**D**) The NECTIN4/NECTIN4-AS1 locus aligned to regulatory elements, as in (A). p53 ChIP-seq peaks are indicated by green arrows. (**E**) RT-qPCR analysis of relative NECTIN4 and NECTIN4-AS1 expression across the hTERT-RPE1 cell panel. (**F**) Relative expression of NECTIN4 and ALDH3A1 in p53WT/p53MUT isogenic lines in the CRC panel described in Figure 1, downloaded from (GSE13886). The units on the Y-axis represent fold-induction. The RT-qPCR results shown are representative of a minimum of two independent experiments.

*ALDH3A1* encodes an aldehyde dehydrogenase, a class of enzymes that are involved in the detoxification of reactive aldehydes that can trigger the production of reactive oxygen species. ALDH3A1 was recently shown to confer resistance to oxidative stress by modulating the DNA damage response [60]. A major p53 ChIP-seq peak was found approximately 5 kb upstream of the annotated first *ALDH3A1* exon (Figure 8A). However, an alternative transcription start site identified by the FANTOM5 consortium [61] was aligned with a regulatory region defined by H3K27 acetylation and the p53 ChIP-seq peak (Figure 8A). Inspection of the sequence immediately upstream of this putative transcription start site revealed a 18/20 match to the consensus p53 response element (Figure 8B). RT-qPCR expression analysis in the hTERT-RPE1 cell panel showed a genotype-dependent pattern virtually identical to that observed in the RNA-seq data, with >10-fold induction by nutlin-3a in unmodified cells (Figure 8C). Notably, the induction of *ALDH3A1* by nutlin-3a was completely eliminated in the *TP53^+/A276P^* cells, suggesting a predominant effect of the p53^A276P^ protein at this locus compared to the canonical targets (Figure 7A).

NECTIN4 (alternatively known as poliovirus receptor-like 4, PVRL4) is an immunoglobulin-like molecule involved in cell-cell adhesion [62]. Three distinct p53 ChIP-seq peaks were apparent at the *NECTIN4* locus (Figure 8D). A minor peak was positioned just upstream of the first coding exon, a second peak was located in the first intron, just upstream of an antisense RNA called NECTIN4-AS1, and a major intragenic peak was located upstream of exon 5. As was the case with validated p53 target genes, the relative expression of *NECTIN4* mRNA was induced >10-fold by nutlin-3a treatment in unmodified hTERT-RPE1 (Figure 8E). However, the basal expression of *NECTIN4* was modestly elevated in both the *TP53*^−/−^ and *TP53^+/A276P^* cells and suppressed by nutlin-3a treatment. This complex expression pattern was not apparent in the RNA-seq data. The spliced NECTIN4-AS1 transcript was slightly induced by nutlin-3a in the p53-knockout cells, but otherwise unaffected by *TP53* genotype.

The upregulation of *ALDH3A1* and *NECTIN4* by p53 was inconsistent among the cell lines in the CRC panel (Figure 8F). In the archived array data, both genes were expressed at levels 2-fold higher in irradiated SW48 *TP53*-wild type cells compared with isogenic p53-knockouts. *NECTIN4* was most robustly induced in *TP53*-wild type HCT116 cells, whereas neither gene was induced to the threshold level by IR in RKO cells.

## DISCUSSION

The successful disruption of both wild type *TP53* alleles in HCT116 provided the first isogenic knockout model for the systematic study of human p53. Over the past 25 years, this model has yielded a wealth of information regarding the cell-autonomous roles of p53 and the vulnerabilities of p53-deficient cancer cells. Nonetheless, our data suggest that the robust growth of the parent cell line was facilitated by an attenuation of p53 activity during the evolution of the original tumor. The increase in senescence and growth inhibition caused by restoration of p53 function in DLD-1 cells may be an indication that these phenotypes are particularly important suppressors of tumorigenesis in CRC epithelia. This conclusion is supported by observations of high levels of senescence in CRC precursor lesions that typically retain *TP53* [63, 64].

Our rationale for studying p53 in hTERT-RPE1 was that *in vitro* immortalization by overexpression of telomerase might have spared the p53 pathway from negative selection that would otherwise occur during *in vivo* tumor evolution. The prediction that these non-cancer cells would retain particularly robust p53 phenotypes was generally supported by the data reported here. Cell growth and survival were increased in cells that were p53-deficient, and downstream transcriptional targets of p53 were highly induced after DNA damage or MDM2 inhibition. A broad range of p53-dependent phenotypes, originally observed in disparate model systems, could be elicited in hTERT-RPE1, suggesting that the complex pathways upstream and downstream of p53 are largely intact and highly sensitive to perturbation.

An important consequence of loss of p53 function is resistance to therapy, particularly radioresistance. A causal association between the mutational inactivation of p53 and decreased radiation sensitivity has been supported by clinical studies [65–67] and preclinical animal models [68, 69]. In contrast, studies of the relationship between p53 status and survival after IR treatment performed in diverse cancer cell line panels have been considerably less definitive [65, 70]. While a sensitizing effect of p53 has recently been observed in several isogenic systems generated by gene editing [71, 72], such effects have been consistently absent in the original HCT116 isogenic cell pair, whether assayed *in vitro* [3] or *in vivo* [2]. The clear radioresistant phenotype of p53-deficient hTERT-RPE1 suggests that this unique cell line will be useful for modeling the effects of IR and other DNA damaging therapies.

A *TP53*-mutant subclone in the hTERT-RPE1 cell population was an unexpected finding. A previous analysis of amino acid substitutions in the A276 position by Reaz et al*.* [73] revealed that this residue could be replaced by serine (S) or phenylalanine (F) with only subtle effects on promoter selection and transcription. In contrast, our data indicate that the substitution of proline (P) in this position created a mutant p53 protein that was not merely transcriptionally inactive, but could exert strong dominant negative effects on transcriptional transactivation by the co-expressed wild type protein. The A276P mutation occurs at low frequency in breast and ovarian cancers, implying that there is selective pressure for expansion of this particular mutation during tumorigenesis. We speculate that this potent mutation arose under selection for p53 loss-of-function in the hTERT-RPE1 population.

It is unclear how the p53^A276P^ mutation initially arose in the hTERT-RPE1 cell line, which is known to be genetically stable. No other alterations in the region of *TP53* were observed. The Cas9 protein from *S. pyogenes* that was employed in our knockout vector most commonly generates small, localized insertions and deletions (indels), with larger deletions observed at some target sites [74]. Based on the well-described patterns of Cas9-mediated mutagenesis, it would seem improbable that the A276P mutation was an off-target product of gene editing. The expanded cell population was found to be clonally heterozygous, indicating that the mutation must have been present among the cell population from which the subclones were derived. An analysis of short tandem repeats confirmed that this clone was indeed derived from hTERT-RPE1, ruling out the possibility of contamination by cancer cells. It is therefore likely that this mutation arose *de novo* during routine cell culture.

It may seem intuitively plausible that driver mutations can arise spontaneously during serial passage of large cell populations, but several studies suggest that this in fact occurs very rarely. Jones et al. [75] found that 287 of 289 mutations discovered in human colon cancer xenografts and cancer-derived cell lines were present in the original primary tumor samples. A follow up analysis by Solomon et al. of a separate set of samples and a review of the published literature also found no evidence of artifactual driver alterations caused by *ex vivo* cell culture [76]. These studies should dispel the misconception that there is significant selection for oncogenic mutations and loss of tumor suppressor genes during the serial maintenance of cancer cell cultures. It could be interesting to determine whether deep sequencing approaches can detect *TP53* mutations, or other driver alterations, that arise and expand under selection in hTERT-RPE1 cell populations.

The suppression of hTERT-RPE1 growth by p53 was likely mediated, at least in part, by the cyclin-dependent kinase inhibitor p21^WAF1/CIP1^, which was expressed at a higher basal level in wild type cells compared with the isogenic p53 knockout line (Figures 3B, 4D). The detection of chromatin-associated p53 tetramers in the absence of exogenous DNA damage (Figure 5B) further supports an active role for p53 in the suppression of unperturbed cell growth in this model. Notably, p53-deficiency in HCT116 does relieve growth inhibition, but this phenotype is dependent on an increase in angiogenesis and is therefore only expressed *in vivo*, in cell-derived xenografts [77].

The transcription-independent roles of p53 are increasingly coming into focus [78, 79]. The role of cytoplasmic p53 on Ca^2+^ flux, first identified by Giorgi et al. [31], has been heretofore associated with apoptotic stimuli. Interestingly, a controlling effect of p53 on Ca^2+^ homeostasis was readily apparent in hTERT-RPE1 the absence of exogenous stressors. The ability of p53 to control Ca^2+^ flux is a function that is lost in the hotspot p53 mutants R175H and R273H*.* [31]. Our results suggest that p53^A276P^, a mutant found in far fewer cancers, is similarly defective. We observed a bimodal distribution of Ca^2+^ content among the p53-knockout and, to a somewhat lesser extent, in the *TP53^+/A276^* cell populations (Figure 6C). The basis for this interesting distribution is unclear. During its catalytic cycle, SERCA is known to function in two distinct structural and biochemical states that differ in their affinity for Ca^2+^ [80]. It is possible that the interaction between SERCA and p53 favors the transition between these two states, which is less efficient in the absence of p53. Alternatively, this bimodal distribution may simply reflect the dynamic changes in intracellular calcium that are known to occur during the cell division cycle, in which case the effect of p53 on SERCA would be indirect. Additional investigation is needed to determine how the regulation of p53 affects SERCA at the single-cell level.

The maintenance of tissue homeostasis by the p53 pathway involves the concerted activities of numerous upstream regulators and downstream effectors. Arguably, the full complexity of this expansive signaling network is unlikely to be captured by any single model system. Complementary studies of different *in vitro* and *in vivo* models, including the immortalized system described here, will undoubtably be needed to form a more complete understanding of p53-mediated tumor suppression. To our knowledge the genes identified in this study, *ALDH3A1* and *NECTIN4*, have not previously been described as direct targets of p53. While prior studies have identified p53 binding sites in and around these loci, we explicitly demonstrate that *ALDH3A1* and *NECTIN4* transcripts can be induced in a p53-dependent manner and suppressed by a dominant-negative p53 variant. We find it interesting that the extent of dominant-negative inhibition varied among different target loci. While MDM2 expression was only partially suppressed in *TP53^+/A276P^* cells, *ALDH3A1* appeared to be completely inhibited by p53^A276P^. More study will be needed to definitively validate the p53-responsiveness of these specific promoter elements *in vitro*, to understand why distinct promoters are differentially affected by different p53 mutants, and to determine the *in vivo* context in which the regulation of these genes by p53 might be relevant to health and disease.

*ALDH3A1* and *NECTIN4* have been previously linked to cancer pathogenesis. Like other members of the aldehyde dehydrogenase superfamily, ALDH3A1 is a multifunctional protein that plays a cytoprotective role under conditions of oxidative stress [60, 81]. A recent study has associated *ALDH3A1* expression with lung cancer metastasis and poor prognosis [82], perhaps challenging its presumed role in homeostasis. NECTIN4 is involved in forming adherens junctions that connect cells to one another and to the extracellular matrix [62, 83]. In addition, NECTIN4 acts as a stimulatory co-receptor for the feedback inhibition of SOCS1 in the JAK2–STAT5a signaling pathway [84]. Expressed at low levels in a variety of normal tissues, NECTIN4 is tumor surface antigen that is highly expressed in subsets of breast, lung, pancreatic and ovarian cancers, and in a majority of urothelial cancers. Notably, NECTIN4 is the target of the antibody-drug conjugate (ADC) enfortumab vedotin, which was USFDA-approved in 2019 for patients with locally advanced or metastatic urothelial carcinoma [85, 86]. Further exploration of ALDH3A1 and NECTIN4 and their regulation by p53 could yield useful insights into the potential roles in tumorigenesis and/or rational drug combinations.

## MATERIALS AND METHODS

### Cell lines and cell culture

A puromycin-sensitive derivative of hTERT-RPE1 was a gift from Andrew Holland. Cells were routinely grown at 37°C in 5% CO_2_ in DMEM/F12 supplemented with 6% fetal bovine serum (FBS) and penicillin/streptomycin. The parental cell line and all derivatives were authenticated by Short Tandem Repeat profiling and tested for the presence of mycoplasma at the Johns Hopkins Genomic Resources Core Facility. CRC cell lines were directly obtained from the Genetic Resources Core Facility Cell Center at Johns Hopkins and grown in McCoy’s 5A supplemented with 6% FBS and penicillin/streptomycin.

### Senescence-associated β-galactosidase staining

Cells in monolayer cultures were fixed with 0.5% glutaraldehyde and stained by the method described by Dimri et al*.* [87]. The results shown are representative of two independent experiments.

### Generation of *hTERT-RPE1* TP53^−/−^ cells

For the disruption of TP53 exon 7, an oligonucleotide duplex encoding the CRISPR guide sequence 5-GCATGGGCGGCATGAACCGG-3 was directly cloned into the plasmid vector pSpCas9(BB)-2A-Puro (pX459) V2.0, a gift from Feng Zhang (Addgene #62988). The resulting plasmid was introduced into hTERT-RPE1 by transfection with Lipofectamine 3000 (ThermoFisher Scientific). Following 4 d of selection in 2 µg/ml puromycin, the remaining cells were plated to limiting dilution in 96-well plates. Individual subclones were expanded and screened by PCR, using the forward primer 5′-CTCCTAGGTTGGCTCTGACTGT-3′ and the reverse primer 5′-AAACTGAGTGGGAGCAGTAAGG-3′. Genetic disruption of both alleles was assessed by Sanger sequencing followed by analysis with Inference of CRISPR Edits software (Synthego). The deletion of *TP53* exon 1 was accomplished by a similar approach. The flanking guides 5′-TAGTATCTACGGCACCAGGT-3′ and 5′-TCAGCTCGGGAAAATCGCTG-3′ were designed to create a 385 bp deletion that included the entire exon. The expected deletion was identified in multiple subclones by PCR with the forward primer 5′-CTCCAAAATGATTTCCACCAAT-3′ and the reverse primer 5′-ACTTTGAGTTCGGATGGTCCTA-3′. For all knockout clones, the loss of p53 expression was confirmed by western blot. Following puromycin selection, subclones were isolated by limiting dilution. Multiple knockout clones were identified by western blot and confirmed by Sanger sequencing of exon 7 or PCR across the exon 1 deletion.

### Identification of the TP53 A276P mutation

Each *TP53* exon in the clone that overexpressed p53 was amplified by PCR and sequenced. A single mutation was identified in exon 8, which was amplified by the forward primer 5′-CTTAGGCTCCAGAAAGGACAAG-3′ and the reverse primer 5′-AGAGGCAAGGAAAGGTGATAAA-3′.

### Western blots, antibodies and cell fractionation

Protein lysates were prepared in RIPA buffer (Cell Signal Technologies), resolved on Bolt Bis-Tris minigels (ThermoFisher Scientific) and transferred to PVDF membranes (MilliporeSigma). Antibodies for the detection of p53 (DO-1) and MDM2 (SMP14) were obtained from Santa Cruz Biotechnology. Antibodies against p21^WAF1/CIP1^ (12D1) and H3K9me3 (D4W1U) phospho-Chk2 (T68, polyclonal), phospho-p53 (S15, 16G8), HSP90 (C45G5), ERp72 (D70D12) and histone H3 (D1H2) were purchased from Cell Signaling Technology. The isolation of ubiquitinated proteins was performed with the Signal-Seeker Ubiquitin Enrichment kit (Cytoskeleton). Cell fractionation was performed with the Qproteome Cell Compartment kit (Qiagen).

### Glutaraldehyde crosslinking

Multimeric forms of p53 were stabilized by glutaraldehyde crosslinking, as previously described [88]. Briefly, cells were lysed with a buffer containing 0.5% NP-40 substitute. Lysates were brought to a final concentration of glutaraldehyde of 0.01% or 0.02% and incubated on ice for 20 min. The crosslinking reaction was stopped with 1X Bolt sample buffer (ThermoFisher Scientific) and proteins were resolved by gel electrophoresis followed by a western blot, as described above.

### Drug treatments

The MDM2 inhibitor nutlin-3a and the proteasome inhibitor MG-132 were purchased from Enzo Life Sciences, dissolved in DMSO and used at final concentrations of 10 µM and 20 µM respectively. The SERCA inhibitor thapsigargin and doxorubicin were purchased from Cell Signaling Technology and dissolved in DMSO. Cycloheximide, used for the assessment of protein stability, was purchased as a ready-made solution (Sigma Aldrich) and used at a final concentration of 100 µg/ml. Phorbol-12-myristate-13-acetate (PMA) was also obtained from Sigma Aldrich, dissolved in ethanol, and used at a final concentration of 100 ng/ml.

### Clonogenic survival assay

For the assessment of clonogenic survival following irradiation, 500–1500 cells were plated to 10 cm cell culture dishes in triplicate. Following a 16–20 h incubation to allow cell attachment, plates were exposed to measured doses of X-rays delivered with a MuliRad225 (Faxitron) and returned to the incubator for 14 d. Colonies were fixed and stained with 0.2% crystal violet in methanol, and colonies containing >50 cells were counted on a plate scanner (Interscience).

### Imaging of subcellular Ca^2+^

Cells were transfected with the plasmid pcDNA3_erGAP2 (a gift from Teresa Alonso and Javier García-Sancho, Addgene #78120), which encodes a low affinity fluorescent calcium biosensor, based on a GFP-aequorin fusion protein (GAP), targeted to the ER. A similar plasmid, pcDNA3_nucGAP (a gift from Teresa Alonso, Addgene #78736) was used to assess nuclear Ca^2+^. Dual-excitation imaging of GAP-expressing cells were performed on a fluorescent microscope equipped with 403 and 470 nm excitation filters.

### Analysis of gene expression

The induction of p53 target genes was assessed by quantitative reverse transcription PCR (RT-qPCR)*.* Total RNA was extracted from subconfluent cells with the Monarch total RNA purification kit (NEB). Reverse transcription and PCR amplification were performed with the Luna One-Step RT-qPCR kit (NEB). Real time PCR amplification was performed on a BioRad CFX96 Real-Time PCR detection system using the primer sets listed in Table 2. RT-qPCR results were cross-referenced with publicly available p53 binding data, as described below.

**Table 2:** Oligonucleotide primers used for RT-qPCR

*CDKN1A (p21^WAF1/CIP1^)*	Forward	AGGTGGACCTGGAGACTCTCAG
	Reverse	TCCTCTTGGAGAAGATCAGCCG
*FDXR*	Forward	TCTTATACCCAATGCTGCTGAG
	Reverse	TCACTAGACTGGAGGGTGTC
*MDM2 (HDM2)*	Forward	GAGAGCAATTAGTGAGACAGAAGA
	Reverse	GCTTTCATCAAAGGAAAGGGAAA
*GAPDH*	Forward	GAGTCAACGGATTTGGTCGT
	Reverse	TTGATTTTGGAGGGATCTCG
*NECTIN4 (PVRL4)*	Forward	GCATCTACGTCTGCCATGTCAG
	Reverse	CTGACACTAGGTCCACCTGCTT
*NECTIN4-AS1*	Forward	CTGGGAATCTCTGTCAGGGC
	Reverse	GTCACTGGGTCTGGCTGTC
*ALDH3A1*	Forward	GCTACATAGCCCCCACCATC
	Reverse	GAACATGTAGAGGGCCAGGG

### RNA-seq analysis

Total RNA was extracted from each cell line, either untreated or following treatment with 10 µM nutlin-3a. Poly(A) selection, library preparation and 2 × 150 bp Illumina sequencing were performed by Azenta Life Sciences. A total of 462,496,793 reads encompassing 138,751 Mb were obtained from the six samples (single replicates of each). Reads were imported into Geneious Prime (Version 2020.0) for processing. Reads were first paired and trimmed with BBDuk, then mapped to Hg38 with the Geneious RNA Mapper. For the calculation of normalized expression levels in transcripts per million, ambiguously mapped reads were counted as partial matches. Differential expression between samples was determined in a pairwise fashion using the median of gene expression ratios, as described [89]. The normalized read counts across samples were used to generate z-scores for each row. Genes that met the specified criteria were clustered by the complete linkage method using the Heatmapper online application (http://heatmapper.ca/expression/). Distance between rows was measured by the Spearman Rank Correlation.

### Analysis of genomic regulatory elements

Histone H3K27Ac and ChIP-seq ReMap data sets generated by the ENCODE consortium [5] and Cap Analysis of Gene Expression (CAGE) data generated by the FANTOM5 consortium [90] were visualized with the BLAST-Like Alignment Tool (BLAT) on the UCSC Genome Browser. ReMap is a comprehensive resource that aggregates transcription factor binding sites from a wide array of publicly available ChIP-seq experiments. The pipeline used by ReMap is designed to uniformly process and annotate ChIP-seq data across multiple datasets. The experiments that assessed p53 binding included two conditions: human cells treated with nutlin and untreated controls. Trimmed sequencing reads were typically aligned to the human reference genome hg38 using Bowtie2. Significant peaks representing prospective p53 binding sites were identified using MACS2 with an FDR threshold of 0.01. Peaks were compared between treated and untreated conditions to identify differential binding sites; DESeq2 was employed for statistical analysis.

## Abbreviations

ChIP: chromatin immunoprecipitation
CRISPR: clustered regularly interspaced short palindromic repeats
CRC: colorectal cancer
ER: endoplasmic reticulum
FBS: fetal bovine serum
GAP: GFP-aequorin fusion protein
IR: ionizing radiation
PMA: phorbol-12-myristate-13-acetate
RT-qPCR: reverse transcription-quantitative PCR
PKC: calcium-dependent protein kinase C
PMA: phorbol 12-myristate 13-acetate
SERCA: SarcoEndoplasmic Ca^2+^-ATPase
